# Age-Dependent Root Apex Closure in Primary Second Molars

**DOI:** 10.3390/children13050616

**Published:** 2026-04-28

**Authors:** Kenan Cantekin, Fahrettin Kalabalık, Mihriban Güner, Münevver Kılıç

**Affiliations:** 1 Faculty of Dentistry, Sakarya University, Sakarya 54050, Turkey; fahrettinkalabalik@sakarya.edu.tr (F.K.); mihribanguner@sakarya.edu.tr (M.G.); 2 Faculty of Dentistry, Istanbul Okan University, Istanbul 34959, Turkey; 3 Faculty of Dentistry, Biruni University, Istanbul 34015, Turkey; munevverk@biruni.edu.tr

**Keywords:** primary second molars, root apex closure, panoramic radiography, dental development, pediatric dentistry

## Abstract

Background: Primary teeth play a crucial role in guiding permanent dentition; however, data regarding root apex closure in primary molars remain limited. The aim of this study was to evaluate the age-related root apex closure status of primary second molars in children aged 2–7 years using panoramic radiographs and to obtain clinically guiding data for pediatric dental treatment planning. Materials and Methods: This cross-sectional, descriptive, and retrospective study evaluated panoramic radiographs. A total of 1628 panoramic radiographs obtained from 1628 patients were evaluated, each representing one individual with primary second molars. The relationships between age groups, gender, and root apex closure status were analyzed statistically with a significance level set at *p* < 0.05. Results: No root apex closure was observed in either maxillary or mandibular primary second molars in the 2–2.99-year age group. In the 3–3.99-year group, a limited number of closed apices were detected only in mandibular primary second molars. A marked increase in the proportion of closed apices was observed in both jaws in the 4–4.99-year group. In the 5–5.99- and 6–6.99-year groups, root apex closure was completed in the majority of maxillary and mandibular primary second molars. Although statistically significant gender-related differences were detected in certain age groups, these differences were not consistent across all age categories. Conclusions: Root apex closure in primary second molars demonstrates a clear age-dependent pattern between 2 and 7 years of age. The findings are expected to provide clinically relevant guidance for pediatric endodontic treatment planning and contribute to the limited literature regarding root development in primary teeth.

## 1. Introduction

Primary teeth are essential for mastication, phonation, esthetics, and the maintenance of arch integrity, as well as for guiding the eruption of permanent successors. Premature loss or pathological involvement of primary molars may result in space loss, malocclusion, and disturbances in the eruption pattern of permanent teeth. Therefore, preservation of primary teeth until their exfoliation is a fundamental objective in pediatric dentistry.

Primary second molars, in particular, play a critical role in maintaining posterior arch length and guiding the eruption of permanent second premolars. Due to their anatomical complexity and high susceptibility to caries, these teeth frequently require pulp therapy. In such clinical scenarios, the stage of root development—especially root apex closure—constitutes a key determinant in treatment selection, prognosis, and risk of complications such as overfilling or damage to underlying permanent tooth germs.

Root development is a biologically regulated process initiated after crown completion and mediated by Hertwig’s epithelial root sheath. Apex closure represents the final stage of root formation and is influenced by multiple factors, including age, tooth type, genetic background, and environmental conditions. Although extensive research has been conducted on root development and apex closure in permanent teeth, corresponding data for primary dentition remain limited and fragmented.

This lack of age-specific and radiographically validated data regarding apex closure in primary second molars may lead clinicians to rely on subjective judgment rather than evidence-based decision-making during pediatric endodontic procedures.

Despite previous studies investigating root development in primary teeth, there is limited evidence specifically focusing on age-dependent root apex closure patterns in primary second molars based on large-scale panoramic radiographic data. Furthermore, the clinical implications of apex closure timing in this specific tooth group remain insufficiently explored. Therefore, this study aims to address this gap by providing comprehensive data on age-related root apex closure in primary second molars.

The null hypothesis (H0) of this study is that there is no statistically significant association between age and root apex closure status in primary second molars.

## 2. Materials and Methods

### 2.1. Study Design and Population

This cross-sectional, descriptive, and retrospective study was conducted using panoramic radiographs archived in the Department of Pediatric Dentistry, Faculty of Dentistry, Sakarya University. Children aged between 2 and 7 years—representing a mixed population without specific ethnic stratification—at the time of radiographic examination were included. Ethical approval was obtained from the Sakarya University Faculty of Dentistry Ethics Committee (Approval No.: 2025/1-17, Date: 18 September 2025).

#### 2.1.1. Sample Size

A total of 1628 panoramic radiographs obtained from 1628 patients were evaluated, each representing one individual with primary second molars.

#### 2.1.2. Inclusion Criteria

- Children aged between 2 and 7 years;
- Availability of high-quality panoramic radiographs;
- Presence of primary second molars.

#### 2.1.3. Exclusion Criteria

- Patients with systemic diseases affecting dental development;
- Poor-quality or non-diagnostic radiographs;
- Missing or unidentifiable primary second molars.

### 2.2. Data Collection

Demographic data (age and gender) were obtained from the patient record system. Chronological age was calculated by subtracting the date of birth from the date of radiographic acquisition, and values obtained in months were converted into decimal years.

### 2.3. Radiographic Assessment

Maxillary and mandibular primary second molars were evaluated. The apical status of each tooth was assessed radiographically and classified as either open apex or closed apex. Teeth presenting with at least one root exhibiting apical openness were classified as “open apex teeth”, whereas teeth with all roots demonstrating complete apex closure were classified as “closed apex teeth”.

In maxillary primary second molars, mesiobuccal, distobuccal, and palatal roots were evaluated separately; in mandibular primary second molars, mesial and distal roots were assessed individually. In cases of uncertainty regarding apex closure, the evaluation was confirmed by consultation with a second experienced pediatric dentist.

### 2.4. Observer Reliability

Radiographic evaluations were performed by a single investigator. To assess intra-observer reliability, a subset of randomly selected radiographs was re-evaluated after a two-week interval, demonstrating high agreement (kappa > 0.90).

### 2.5. Age Groups

Participants were divided into five age groups according to chronological age: 2–2.99, 3–3.99, 4–4.99, 5–5.99, and 6–6.99 years. In each age group, the open and closed apex status of primary second molars was analyzed.

### 2.6. Statistical Analysis

Statistical analyses were performed using IBM SPSS Statistics version 26.0 (IBM Corp., Chicago, IL, USA). Associations between root apex closure status and age groups and gender were evaluated using Pearson’s chi-square test and Fisher’s exact test when appropriate. Statistical significance was set at *p* < 0.05.

## 3. Results

The age and gender distribution of participants is presented in Table 1. The sample consisted of 52.9% females and 47.1% males, with the largest number of teeth observed in the 6–6.99-year age group (Table 1).

In the 2–2.99-year age group, no root apex closure was observed in either maxillary or mandibular primary second molars. In the 3–3.99-year group, a limited number of closed apices were detected only in mandibular primary second molars. In the 4–4.99-year group, a marked increase in closed apex proportions was observed in both jaws. In the 5–5.99- and 6–6.99-year groups, root apex closure was completed in the majority of maxillary and mandibular primary second molars (Table 2).

Root-based evaluation revealed that, in maxillary primary second molars, apex closure occurred earlier in the mesiobuccal and distobuccal roots compared with the palatal root. In mandibular primary second molars, mesial roots demonstrated earlier closure than distal roots. In all groups aged five years and older, nearly all evaluated roots exhibited complete apex closure (Table 3).

Gender-based analysis demonstrated no significant difference in maxillary primary second molars in the 4–4.99-year group; however, in the 5–5.99-year group, males exhibited a significantly higher rate of open apices compared with females (*p* < 0.05). No significant differences were detected in other age groups (Table 4).

In mandibular primary second molars, no significant gender-related differences were observed in the 3–3.99- and 4–4.99-year groups. In age groups five years and older, complete root apex closure was observed in all individuals (Table 5).

## 4. Discussion

Dental development and apex closure timing represent important biological indicators that directly influence clinical decision-making in pediatric and endodontic treatment planning [1,2,3]. The present study evaluated the root apex closure status of primary second molars in children aged 2–7 years using panoramic radiographs and demonstrated a clear age-dependent increase in closed apex rates.

The absence of root apex closure in the 2–3-year age group indicates that root development in primary second molars is not yet completed at these ages. Conversely, a marked increase in closed apices was observed after the age of four, and root apex closure was completed in the majority of teeth in children aged five years and older. These findings are consistent with the general pattern reported in dental maturation studies describing root development as a progressive and age-dependent process [4,5,6,7,8].

Most previous studies on apex closure timing have focused on permanent teeth. Methods developed for dental maturation and age estimation predominantly rely on mineralization and root development stages of permanent dentition [4,5,6]. Radiographic studies conducted in various populations have primarily examined apical development of permanent molars and incisors, describing age-related closure patterns in detail [9,10,11]. In contrast, developmental data regarding primary teeth remain limited, possibly due to the early onset of physiological root resorption and their temporary nature [1,3].

Primary second molars play a critical role in maintaining posterior arch stability and guiding eruption of permanent second premolars [1,12]. These teeth frequently require pulpotomy or pulpectomy due to deep carious lesions, and the stage of root development significantly influences treatment choice and prognosis [2,13,14,15]. However, the limited availability of age-specific radiographic data on root apex closure in primary second molars often results in clinical decisions being largely experience-based. Therefore, this study aimed to provide age-specific evaluation of root development in these teeth.

Root-based findings in this study—earlier closure of buccal roots compared with palatal roots in maxillary molars, and earlier closure of mesial roots compared with distal roots in mandibular molars—are consistent with previous morphological and developmental investigations [3,5,16]. These findings suggest that root development may vary not only according to tooth type but also according to root morphology and functional load distribution [6,8,17].

Dental development is influenced by genetic, epigenetic, and environmental factors, leading to population variability [8,18,19]. Gender-based analysis revealed a statistically significant difference only in the 5–5.99-year group for maxillary molars, with males demonstrating higher rates of open apices. However, no consistent gender-related differences were observed across all age groups or in mandibular molars. Previous studies have reported earlier dental development in females compared with males [20,21]. However, Liversidge [5] and Liversidge and Chaillet [22] emphasized that gender-related differences in dental development are not consistent across all tooth types and age ranges. The limited and age-specific gender differences observed in this study appear consistent with this heterogeneous pattern.

From a clinical perspective, knowledge of root apex closure status in primary second molars enhances predictability in decisions regarding pulpotomy, pulpectomy, or extraction [2,12]. Although guidelines from the International Association of Dental Traumatology (IADT) and the American Academy of Pediatric Dentistry (AAPD) emphasize consideration of root development in treatment planning, specific age ranges for primary second molars are not clearly defined [2,7]. Therefore, the present findings may provide practical guidance for clinical decision-making.

This study has certain limitations. The retrospective design and reliance on panoramic radiographs offer lower spatial resolution compared with cone-beam computed tomography (CBCT) for apical assessment [14,23]. However, the routine use and lower radiation exposure associated with panoramic imaging in pediatric patients enhance the clinical applicability of the findings [14,19]. The findings of this study reject the null hypothesis, demonstrating a significant association between age and root apex closure.

### 4.1. Limitations of the Study

This study has several limitations. First, its retrospective design may introduce selection bias and limit the control over data collection. Second, the use of panoramic radiographs may not provide the same level of detail as three-dimensional imaging modalities, potentially affecting the accuracy of apex closure assessment. Third, the study was conducted using data from a single center, which may limit the generalizability of the findings to broader populations. Despite these limitations, the large sample size represents a significant strength of the study.

### 4.2. Future Research Directions

Future studies should consider longitudinal designs to better understand the dynamic process of root development and apex closure in primary teeth. Additionally, the use of advanced imaging techniques such as cone-beam computed tomography (CBCT) may provide more detailed and precise evaluations. Expanding research to multi-center populations and different ethnic groups would also enhance the generalizability of findings.

## 5. Conclusions

The findings of this study demonstrate that root apex closure in primary second molars exhibits a clear age-dependent pattern in children aged 2–7 years. These results provide clinically relevant information that may assist pediatric dentists in treatment planning and timing of interventions. The study contributes to the existing literature by offering comprehensive data on apex closure patterns in this specific tooth group [6,16,22].

## Figures and Tables

**Table 1:** Age and gender distribution of the participants.

Age Group (Years)	Gender				Total	
	Female		Male			
	*N*	%	*N*	%	*N*	%
2–2.99	20	1.2%	8	0.5%	28	1.7%
3–3.99	64	3.9%	56	3.4%	120	7.4%
4–4.99	164	10.1%	178	10.9%	342	21.0%
5–5.99	294	18.1%	250	15.4%	544	33.4%
6–6.99	320	19.7%	274	16.8%	594	36.5%
Total	862	52.9%	766	47.1%	1628	100%

**Table 2:** Proportion of closed apex for primary second maxillary and mandibular molars by age group.

Age Groups (Years)	Maxillary Molars		Mandibular Molars	
	*N*	%	*N*	%
2–2.99	0	0.0%	0	0.0%
3–3.99	0	0.0%	4	0.3%
4–4.99	100	6.2%	232	14.9%
5–5.99	533	33.2%	527	33.8%
6–6.99	574	35.8%	557	35.7%

**Table 3:** Proportion of closed root apices for each root of primary second molars by age group.

Age Group (Years)	Maxillary Molar						Mandibular Molar			
	Mesiobuccal Root		Distobuccal Root		Palatal Root		Mesial Root		Distal Root	
	*N*	*%*	*N*	%	*N*	%	*N*	%	*N*	%
2–2.99	0	0.0%	0	0.0%	0	0.0%	0	0.0%	0	0.0%
3–3.99	38	31.7%	38	31.7%	0	0.0%	39	33.3%	5	4.3%
4–4.99	321	94.1%	316	92.7%	101	29.6%	314	94.9%	233	70.4%
5–5.99	541	100%	541	100%	533	98.5%	528	100%	527	100%
6–6.99	574	100%	574	100%	574	100%	557	100%	559	100%

**Table 4:** Proportion of open and closed apex for primary second maxillary molars by age group according to gender.

Age Group (Years)		Gender		*p*	Total
		Female *N* (%)	Male *N* (%)		
2–2.99	Open Apex	20 (100.0%)	8 (100.0%)	-	28 (100.0%)
	Closed Apex	0 (0.0%)	0 (0.0%)		0 (0.0%)
3–3.99	Open Apex	64 (100.0%)	56 (100.0%)	-	120 (100.0%)
	Closed Apex	0 (0.0%)	0 (0.0%)		0 (0.0%)
4–4.99 ^x^	Open Apex	119 (73.0%)	122 (68.5%)	0.36	241 (70.7%)
	Closed Apex	44 (27.0%)	56 (31.5%)		100 (29.3%)
5–5.99 ^y^	Open Apex	0 (0.0%)	8 (3.2%)	**0.002** *	8 (1.5%)
	Closed Apex	291 (100.0%)	242 (96.8%)		533 (98.5%)
6–6.99 ^x^	Open Apex	0 (0.0%)	0 (0.0%)	-	0 (0.0%)
	Closed Apex	313 (100.0%)	261 (100.0%)		574 (100.0%)

**^x^** = Chi-square test; **^y^** = Fisher’s exact test; The significance level was set to *p* < 0.05. *: *p* < 0.05.

**Table 5:** Proportion of open and closed apex for primary second mandibular molars by age group according to gender.

Age Group (Years)		Gender		*p*	Total
		Female N (%)	Male N (%)		
2–2.99	Open Apex	20 (100.0%)	8 (100.0%)	-	28 (100.0%)
	Closed Apex	0 (0.0%)	0 (0.0%)		0 (0.0%)
3–3.99 ^y^	Open Apex	60 (95.2%)	53 (98.1%)	0.62	113 (96.6%)
	Closed Apex	3 (4.8%)	1 (1.9%)		4 (3.4%)
4–4.99 ^x^	Open Apex	48 (30.0%)	51 (29.8%)	0.97	99 (29.9%)
	Closed Apex	112 (70.0%)	120 (70.2%)		232 (70.1%)
5–5.99	Open Apex	0 (0.0%)	0 (0.0%)	-	0 (0.0%)
	Closed Apex	283 (100.0%)	244 (100.0%)		527 (100.0%)
6–6.99	Open Apex	0 (0.0%)	0 (0.0%)	-	0 (0.0%)
	Closed Apex	296 (100.0%)	261(100.0%)		557 (100.0%)

**^x^** = Fisher’s exact test; **^y^** = Chi-square test; The significance level was set to *p* < 0.05.

## Data Availability

The data sets used and/or analyzed during the current study are available from the corresponding author on reasonable request. We guarantee that the data will be shared if requested by your journal.

## References

[1] Redwan A.K., Alhazmi H.A., Alharthi S.A., Alharbi J.J. (2024). Parents’ Knowledge and Awareness About the Importance of Primary Teeth and Space Maintainers in Saudi Arabia: A Cross-Sectional Study. Cureus.

[2] Goerig A.C., Camp J.H. (1983). Root canal treatment in primary teeth: A review. Pediatr. Dent..

[3] American Academy of Pediatric Dentistry (2025). Pulp therapy for primary and immature permanent teeth. Reference Manual of Pediatric Dentistry.

[4] Thomas H.F. (1995). Root formation. Int. J. Dev. Biol..

[5] Demirjian A., Goldstein H., Tanner J.M. (1973). A new system of dental age assessment. Hum. Biol..

[6] Liversidge H.M. (2006). Timing of human mandibular tooth formation. Ann. Hum. Biol..

[7] Kurniawan A., Chusida A., Atika N., Gianosa T.K., Solikhin M.D., Margaretha M.S., Utomo H., Marini M.I., Rizky B.N., Prakoeswa B.F.W.R. (2022). The applicable dental age estimation methods for children and adolescents. Int. J. Dent..

[8] Levin L., Day P.F., Hicks L., O’COnnell A., Fouad A.F., Bourguignon C., Abbott P.V. (2020). International Association of Dental Traumatology guidelines for the management of traumatic dental injuries: General introduction. Dent. Traumatol..

[9] Dean J.A., Avery D.R., McDonald R.E. (2022). McDonald and Avery’s Dentistry for the Child and Adolescent.

[10] Iandolo A., Pisano M., Scelza G., Abdellatif D., Martina S. (2022). Three-dimensional evaluation of the root apex of permanent maxillary premolars: A multicentric study. Appl. Sci..

[11] Khalaf K., Brook A.H., Smith R.N. (2022). Genetic, epigenetic and environmental factors influence the phenotype of tooth number, size and shape. Genes..

[12] Anees W., Shah F.M., Khan J.N., Jabeen N., Siddique H.M.A.B., Shoro S., Akram S., Ullah I. (2022). Assessment of root apices closure of permanent incisors and first molars in digital orthopantomographs: A pilot study from Pakistan. Pak. J. Med. Health Sci..

[13] Casamassimo P.S., Fields H.W., McTigue D.J., Nowak A.J. (2013). Pediatric Dentistry: Infancy Through Adolescence.

[14] Kalabalık F., Yılmaz N., Aydın E.G., Aytuğar E. (2024). Investigation of root apical closure of first permanent molars using cone-beam computed tomography. J. Dent. Sci..

[15] White S.C., Pharoah M.J. (2019). Oral Radiology: Principles and Interpretation.

[16] Fuks A.B., Casamassimo P.S., Fields H.W., McTigue D.J., Nowak A.J. (2013). Pulp therapy for the primary dentition. Pediatric Dentistry: Infancy Through Adolescence.

[17] Liversidge H.M., Molleson T. (2004). Developing permanent tooth length as an estimate of age. J. Forensic Sci..

[18] Haiter-Neto F., Kurita L.M., Menezes A.V. (2007). Dental maturity as an indicator of chronological age. J. Appl. Oral. Sci..

[19] Dhamo B., Halazonetis D.J., Kuijpers-Jagtman A.M. (2017). Ancestry and dental development: A geographic and genetic perspective. Am. J. Phys. Anthropol..

[20] Valente R.P.A., Lima L.K.G., Bueno J.M., Oliveira M.B., Franco A., Paranhos L.R. (2024). Nicodemo’s method on dental development: A cross-sectional study with 3271 children and adolescents. Braz. Oral. Res..

[21] Ceyhan D., Akdik C., Kırzıoğlu Z. (2019). Çocuk hastalarda odontomaların değerlendirilmesi: Retrospektif-kesitsel bir çalışma. Atatürk Üniv. Diş. Hek. Fak. Derg..

[22] AlQahtani S.J., Hector M.P., Liversidge H.M. (2010). Brief communication: The London atlas of human tooth development and eruption. Am. J. Phys. Anthropol..

[23] Liversidge H.M., Chaillet N., Mörnstad H., Nyström M., Rowlings K., Taylor J., Willems G. (2006). Timing of Demirjian’s tooth formation stages. Ann. Hum. Biol..

